# Supplementary material for: The RRM-mediated RNA binding activity in T. brucei RAP1 is essential for VSG monoallelic expression
Source: Nat Commun. 2023 Mar 22;14:1576. doi: 10.1038/s41467-023-37307-0 (PMC10033678; doi:10.1038/s41467-023-37307-0)
Supplement: Supplementary file 1 — Supplementary information [file 41467_2023_37307_MOESM1_ESM.pdf]

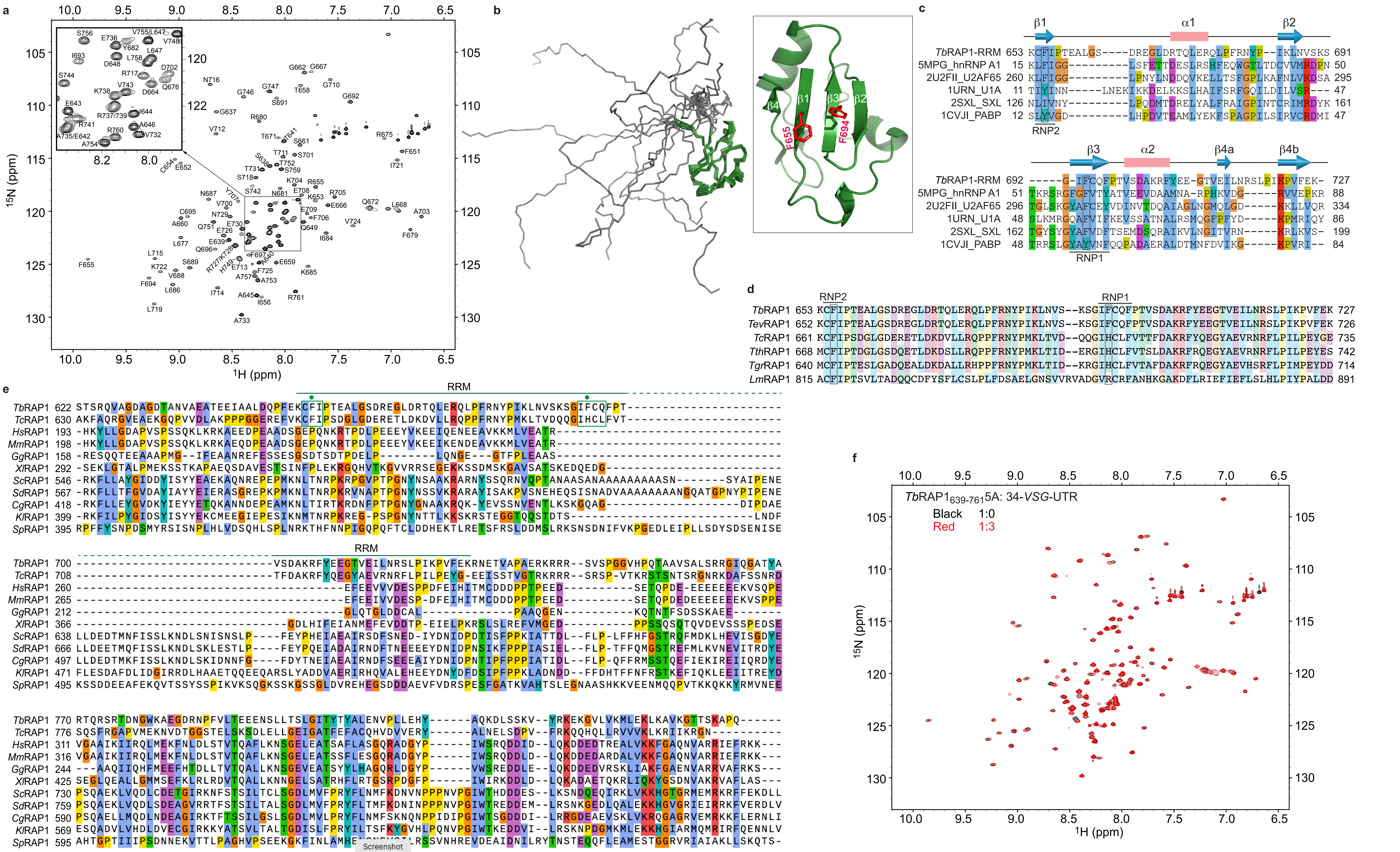

a
^{1}H (ppm)
^{15}N (ppm)
b
F655
F694
β1
β2
β3
β4
c
β1
α1
β2
β3
α2
β4a
β4b
TbRAP1-RRM 653
5MPG_hnRNP A1 15
2U2FII_U2AF65 260
1URN_U1A 11
2SXL_SXL 126
1CVJI_PABP 12
RNP2
RNP1
d
RNP2
RNP1
TbRAP1 653 KCFIPTEALGSDREGLDRTQLERQLPFRNYPIKLNVS--KSGIFCQFPTVSDAKRFYEEGTVEILNRSLPIKPVFEK 727
TevRAP1 652 KCFIPTEALGSDREGLDRTQLERQLPFRNYPIKLNVS--KSGIFCQFPTVSDAKRFYEEGTVEILNRSLPIKPVFEK 726
TcRAP1 661 KCFIPSDGLGDERETLDKDVLLRQPPFRNYPMKLTVD--QQGIHCLFVTTFDAKRFYQEGYAEVRNRFLPILPEYGE 735
TthRAP1 668 MCFIPTDGLGSDQETLDKDSLLRQPPFRNYPMKLTID--ERGIHCLFVTSLDAKRFRQEGYAEVRNRFLPILPEYES 742
TgrRAP1 640 MCFIPTESLGADQEALDKDALLRHPPFRNYPMKLTID--KRGIHCLFVTAFDAKRFRQEGYAEVHSRFLPIMPEYDD 714
LmRAP1 815 ACFIPTSVLTADQQCDFYSFLCSLPLFDSAELGNSVVRVADGVRCRFANHKGAKDFLRIEFIEFLSLHLPIYPALDD 891
e
RRM
TbRAP1
TcRAP1
HsRAP1
MmRAP1
GgRAP1
XlRAP1
ScRAP1
SdRAP1
CgRAP1
KlRAP1
SpRAP1
f
TbRAP1639-761 5A: 34-VSG-UTR
Black 1:0
Red 1:3
^{1}H (ppm)
^{15}N (ppm)

Supplementary Fig. 1. The *Tb*RAP1 RRM module shows varied binding to different RNA substrates. **a** Backbone NMR assignment of *Tb*RAP1$_{639\text{-}761}$. The assignment of each peak is given with residue one-letter code and sequence number. The inset shows assignment details in the central region for clarity. **b** 10 best solution structures of *Tb*RAP1$_{639\text{-}761}$ without restraint violation were superimposed by aligning the RRM module. The N-and C-terminal regions are highly flexible. Inset, one representative structural model of *Tb*RAP1$_{653\text{-}727}$ (RRM only) is shown with the four β strands in the RRM module labelled as β1-β4. The conserved phenylalanine residues (F655 and F694) are shown on β1 and β3, respectively. **c** Sequence alignment of *Tb*RAP1$_{653\text{-}727}$ with 5 representative RRM domains. Each domain is labeled by its PDB ID followed by the molecule name. Secondary structural elements are marked above the alignment. The RNP1 and RNP2 conserved sequence motifs are underlined and the two aromatic residues in RNP1 and RNP2 are boxed. Conserved residues are highlighted. **d** Sequence alignment of the RRM domain in representative Trypanosomatida RAP1s. *Tb*RAP1, *T. brucei* RAP1, Tb427tmp.03.0760 (TriTrypDB ID); *Tev*RAP1, *T. evansi* RAP1, TevSTIB805.11_01.300; *Tc*RAP1, *T. cruzi* RAP1, TcCLB.507093.72; *Tth*RAP1, *T. theileri* RAP1, TM35_000054420; *Tgr*RAP1, *T. grayi* RAP1, DQ04_01601090; *Lm*RAP1, *Leishmania amazonensis* RAP1, LAMA_000481300. Amino acid residues are colored according to ClustalX, and their positions are indicated. The RNP1 and RNP2 motifs are labeled on top of the alignment. Residues aligned to the two key phenylalanine resides in *Tb*RAP1 are boxed. **e** Sequence alignments of *Tb*RAP1-MybLike domain against RAP1 homologs in the same region. *Tb*, *T. brucei*; *Tc*, *T. cruzi*; *Hs, Homo sapiens; Mm, Mus musculus; Gg, Gallus gallus; Xl, Xenopus laevis; Sc, Saccharomyces cerevisiae; Sd, Saccharomyces dairenensis; Cg, Candida glabrata; Kl, Kluyveromyces lactis; Sp, Schizosaccharomyces pombe.* *Tb*RAP1 RRM is indicated by a line on top of the alignment. The conserved RNP1 and RNP2 in *Tb*RAP1 and *Tc*RAP1 are boxed. **f** ^{1}H-^{15}N HSQC NMR spectra of ^{15}N-labeled *Tb*RAP1$_{639\text{–}761}$5A in the absence (black) and presence of 34-*VSG*-UTR oligo in 3× molar excess (red). *Tb*RAP1$_{639\text{–}761}$5A induced chemical shifts similar to those in *Tb*RAP1$_{639\text{-}733}$ but smaller than in *Tb*RAP1$_{639\text{-}761}$.

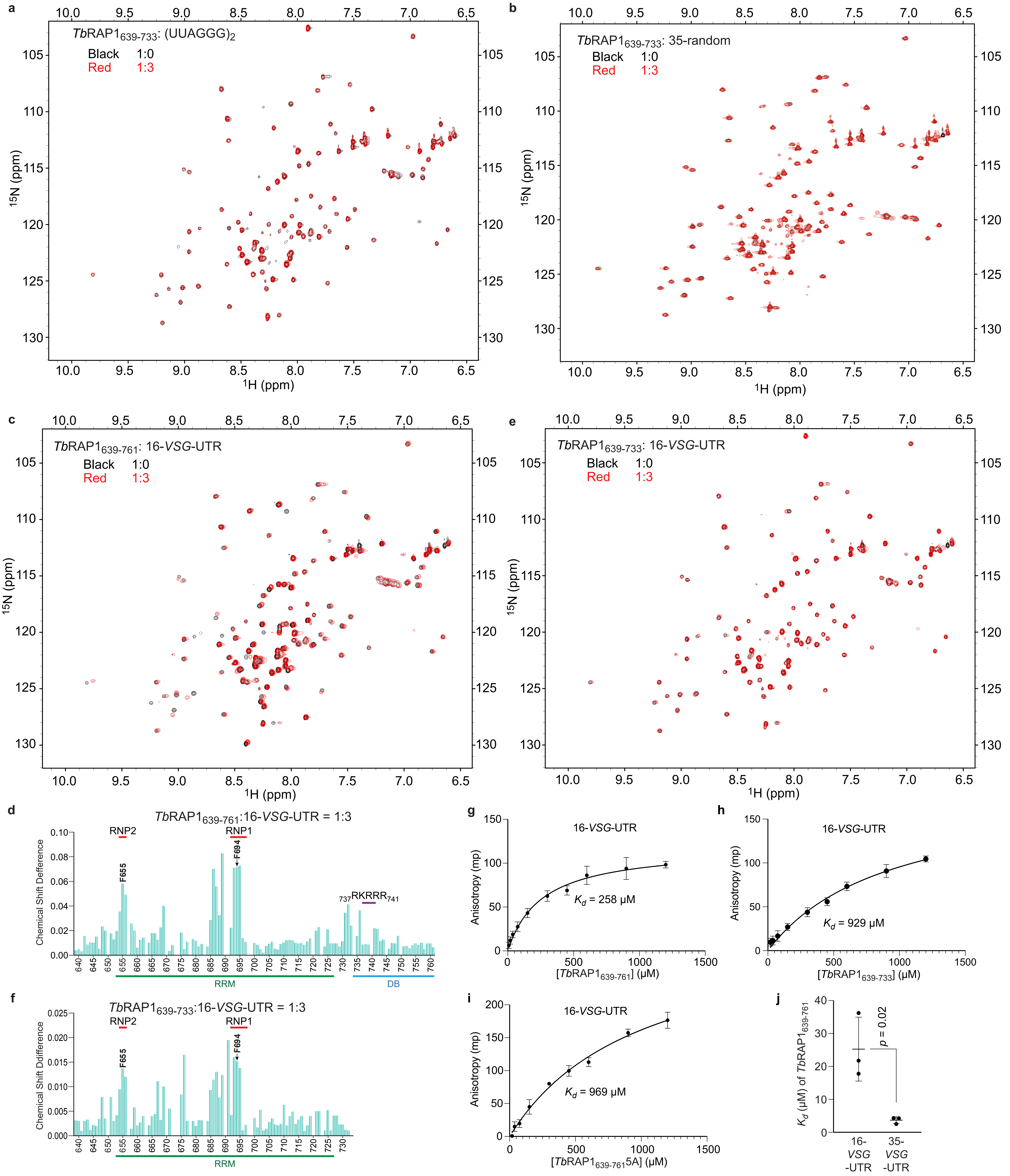
a
TbRAP1639-733: (UUAGGG)2
Black 1:0
Red 1:3
15N (ppm)
1H (ppm)
b
TbRAP1639-733: 35-random
Black 1:0
Red 1:3
c
TbRAP1639-761: 16-VSG-UTR
Black 1:0
Red 1:3
e
TbRAP1639-733: 16-VSG-UTR
Black 1:0
Red 1:3
d
TbRAP1639-761:16-VSG-UTR = 1:3
Chemical Shift Defference
RNP2
RNP1
F655
F694
737RKRRR741
RRM
DB
f
TbRAP1639-733:16-VSG-UTR = 1:3
Chemical Shift Ddifference
RNP2
RNP1
F655
F694
RRM
g
16-VSG-UTR
Anisotropy (mp)
Kd = 258 μM
[TbRAP1639-761] (μM)
h
16-VSG-UTR
Anisotropy (mp)
Kd = 929 μM
[TbRAP1639-733] (μM)
i
16-VSG-UTR
Anisotropy (mp)
Kd = 969 μM
[TbRAP1639-7615A] (μM)
j
Kd (μM) of TbRAP1639-761
p = 0.02
16-VSG-UTR
35-VSG-UTR

Supplementary Fig. 2. *Tb*RAP1 RRM domain has an RNA binding activity. **a, b** ^{1}H-^{15}N HSQC NMR spectra of ^{15}N-labeled *Tb*RAP1$_{639–733}$ in the absence (black) and the presence (red) of (UUAGGG)$_2$ (**a**) and 35-random (**b**). No chemical shifts were observed. **c, e** ^{1}H-^{15}N HSQC NMR spectra of ^{15}N-labeled *Tb*RAP1$_{639–761}$ (**c**) and *Tb*RAP1$_{639–733}$ (**e**) in the absence (black) and presence (red) of 16-*VSG*-UTR. PPM, parts per million. **d, f** Chemical shift differences in NMR titration experiments shown in (**c**) and (**e**), respectively. **g-**i Binding affinity of *Tb*RAP1$_{639–761}$ (**g**), *Tb*RAP1$_{639–733}$ (**h**), and *Tb*RAP1$_{639–761}$5A (**i**) to 16-*VSG*-UTR measured by fluorescent polarization. Binding curves are shown, and K_d values are indicated. Average and standard deviation were calculated from three independent experiments. **j** Binding affinity of *Tb*RAP1$_{639–761}$ to 35-*VSG*-UTR and 16-*VSG*-UTR estimated by EMSA. Average and standard deviation were calculated from three independent experiments. *P* values of two-sided unpaired *t*-tests are shown (35-*VSG*-UTR vs 16-*VSG*-UTR). Error bars in (**g-j**) represent standard deviation. Source data for (**d, f-j**) are provided as a Source Data file.

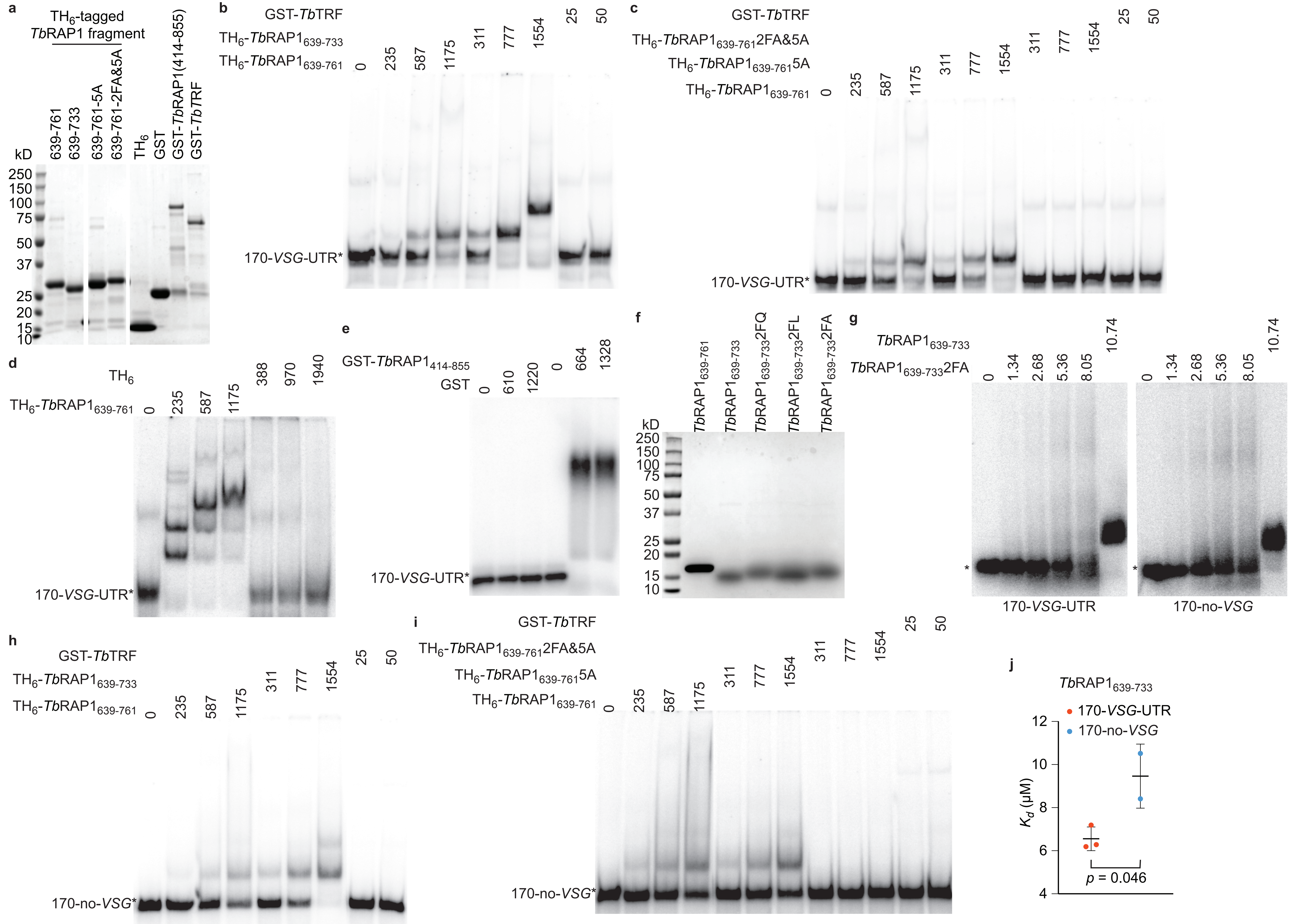
a
TH6-tagged
TbRAP1 fragment
639-761
639-733
639-761-5A
639-761-2FA&5A
TH6
GST
GST-TbRAP1(414-855)
GST-TbTRF
kD
250
150
100
75
50
37
25
20
15
10
b
GST-TbTRF
TH6-TbRAP1639-733
TH6-TbRAP1639-761
0
235
587
1175
311
777
1554
25
50
170-VSG-UTR*
c
GST-TbTRF
TH6-TbRAP1639-7612FA&5A
TH6-TbRAP1639-7615A
TH6-TbRAP1639-761
0
235
587
1175
311
777
1554
311
777
1554
25
50
170-VSG-UTR*
d
TH6
TH6-TbRAP1639-761
0
235
587
1175
388
970
1940
170-VSG-UTR*
e
GST-TbRAP1414-855
GST
0
610
1220
0
664
1328
170-VSG-UTR*
f
kD
TbRAP1639-761
TbRAP1639-733
TbRAP1639-7332FQ
TbRAP1639-7332FL
TbRAP1639-7332FA
250
150
100
75
50
37
25
20
15
10
g
TbRAP1639-733
TbRAP1639-7332FA
0
1.34
2.68
5.36
8.05
10.74
0
1.34
2.68
5.36
8.05
10.74
*
*
170-VSG-UTR
170-no-VSG
h
GST-TbTRF
TH6-TbRAP1639-733
TH6-TbRAP1639-761
0
235
587
1175
311
777
1554
25
50
170-no-VSG*
i
GST-TbTRF
TH6-TbRAP1639-7612FA&5A
TH6-TbRAP1639-7615A
TH6-TbRAP1639-761
0
235
587
1175
311
777
1554
311
777
1554
25
50
170-no-VSG*
j
TbRAP1639-733
170-VSG-UTR
170-no-VSG
Kd (µM)
12
10
8
6
4
p = 0.046

Supplementary Fig. 3. *Tb*RAP1 RRM has an RNA binding activity. **a, f** Partially purified recombinant proteins with tags (**a**) and without the TrxA-His$_6$ (TH$_6$) tag (**f**) were analyzed by polyacrylamide gel electrophoresis. **b-e, g-i** The RNA binding activity of *Tb*RAP1 was analyzed by EMSA experiments. TH$_6$-*Tb*RAP1$_{639\text{-}733}$ (**b, g, h**), TH$_6$-*Tb*RAP1$_{639\text{-}761}$ (**b, c, d, h,** i), TH$_6$-*Tb*RAP1$_{639\text{-}761}$2FA&5A (**c, i**), TH$_6$-*Tb*RAP1$_{639\text{-}761}$5A (**c, i**), TH$_6$ (**d**), GST-*Tb*RAP1$_{414\text{-}855}$ (**e**), GST (**e**), and GST-*Tb*TRF (**b, c, h,** i) were used in EMSA. Radiolabeled 170-*VSG*-UTR (**b-e**) and 170-no-*VSG* (**h, i**) were used as the substrate. **g** Tagless *Tb*RAP1$_{639\text{-}733}$ and *Tb*RAP1$_{639\text{-}761}$2FA were examined for their RNA binding activity in EMSA. Radiolabeled 170-*VSG*-UTR (left) and 170-no-*VSG* (right) were used as the substrate. Asterisks represent free probes. The concentration of protein used (nM) is indicated on top of each lane in (**b-e** & **h-i**) and the concentration of protein used (μM) is indicated in (**g**). Samples were electrophoresed in 5% native polyacrylamide gels (**b, c, d, h, i**) or 0.8% agarose gels (**e, g**) in 0.5 x TBE running buffer. **j** Binding affinities of tagless *Tb*RAP1$_{639\text{-}733}$ to 170-*VSG*-UTR and 170-no-*VSG*. EMSA results were quantified using ImageQuant, and K_d was calculated using GraphPad Prism from two (for 170-no-*VSG*) or three (for 170-*VSG*-UTR) independent experiments. The *p* value of two-sided unpaired *t*-test is shown. Error bars represent standard deviation. Source data are provided as a Source Data file.

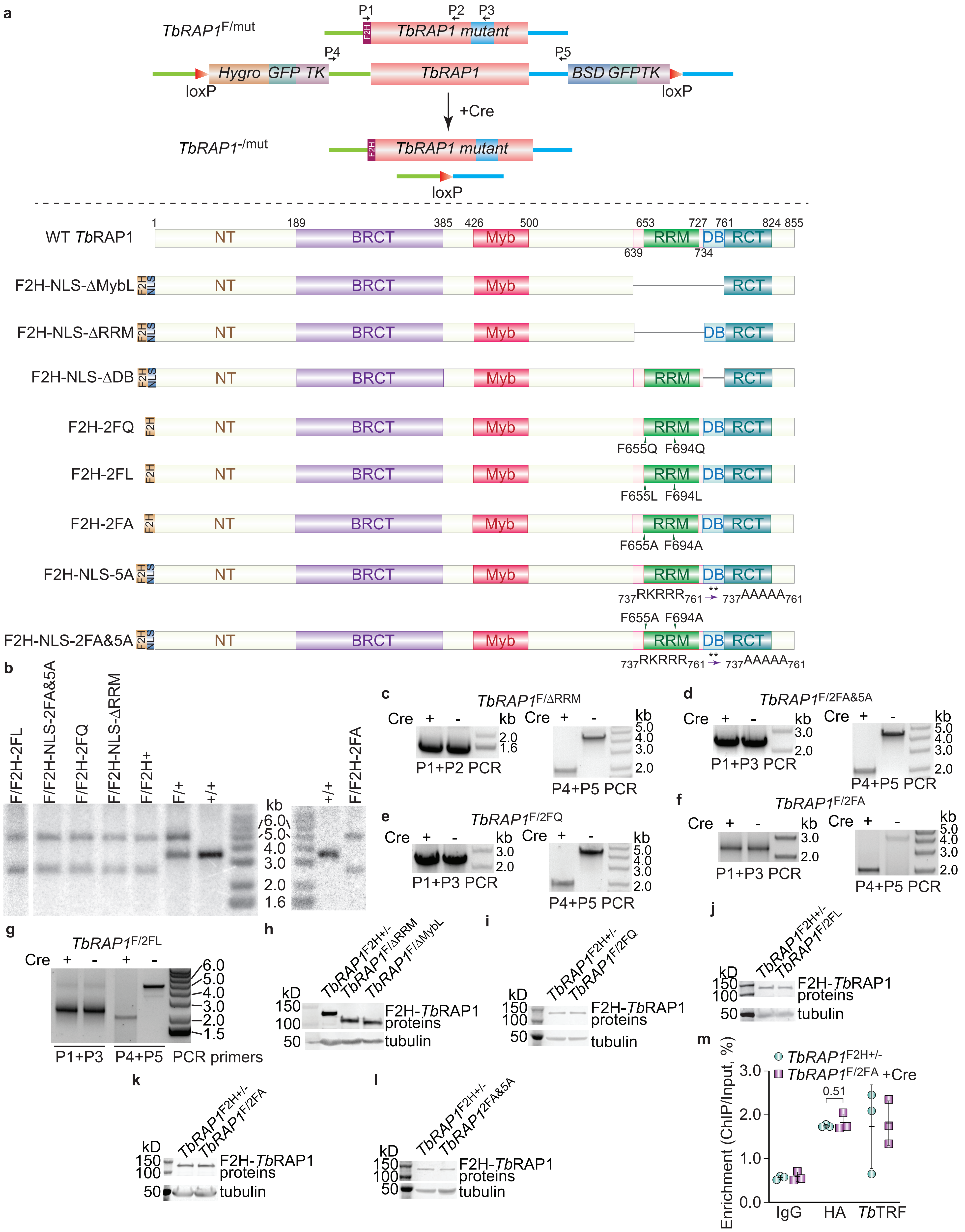
a
TbRAP1F/mut
P1 P2 P3
F2H
TbRAP1 mutant
P4 P5
Hygro GFP TK
TbRAP1
BSD GFPTK
loxP
loxP
+Cre
TbRAP1-/mut
loxP
WT TbRAP1
1 189 385 426 500 653 727 761 824 855
639 734
NT BRCT Myb RRM DB RCT
F2H-NLS-ΔMybL
F2H-NLS-ΔRRM
F2H-NLS-ΔDB
F2H-2FQ
F655Q F694Q
F2H-2FL
F655L F694L
F2H-2FA
F655A F694A
F2H-NLS-5A
737RKRRR761 ** 737AAAAA761
F2H-NLS-2FA&5A
b
F/F2H-2FL
F/F2H-NLS-2FA&5A
F/F2H-2FQ
F/F2H-NLS-ΔRRM
F/F2H+
F/+
+/+
+/+
F/F2H-2FA
kb 6.0 5.0 4.0 3.0 2.0 1.6
c
TbRAP1F/ΔRRM
Cre + - kb
P1+P2 PCR
P4+P5 PCR
d
TbRAP1F/2FA&5A
P1+P3 PCR
P4+P5 PCR
e
TbRAP1F/2FQ
f
TbRAP1F/2FA
g
TbRAP1F/2FL
P1+P3 P4+P5 PCR primers
h
TbRAP1F2H+/- TbRAP1F/ΔRRM TbRAP1F/ΔMybL
kD 150 100 50
F2H-TbRAP1 proteins
tubulin
i
TbRAP1F2H+/- TbRAP1F/2FQ
j
TbRAP1F2H+/- TbRAP1F/2FL
k
TbRAP1F2H+/- TbRAP1F/2FA
l
TbRAP1F2H+/- TbRAP12FA&5A
m
TbRAP1F2H+/-
TbRAP1F/2FA +Cre
Enrichment (ChIP/Input, %)
0.51
IgG HA TbTRF

Supplementary Fig. 4. Generation and validation of *TbRAP1*$^{F/mut}$ strains. **a** Top, a diagram showing the two *TbRAP1* alleles in *TbRAP1*$^{F/mut}$ cells before and after the Cre induction. PCR primers used for validating the deletion of the floxed *TbRAP1* allele after the Cre induction are marked with arrows. Bottom, a diagram showing various *Tb*RAP1 mutants used in this study. **b** Southern analysis to verify the genotype of *TbRAP1*$^{F/mut}$ strains. Genomic DNA isolated from *TbRAP1*$^{F/mut}$ cells were digested with EcoRI. The NT region of the *TbRAP1* gene (Fig. 1c) was used as the probe. The WT *TbRAP1* allele gives a 3.4 kb band, the floxed *TbRAP1* allele gives a 4.7 kb band, and mutant *TbRAP1* alleles give a 2.5 kb band. **c-g** PCR analyses were performed using the indicated primer pairs to confirm the deletion of the floxed *TbRAP1* allele and the retaining of the F2H-tagged mutant allele after the Cre induction in *TbRAP1*$^{F/\Delta RRM}$ (**c**), *TbRAP1*$^{F/2FA\&5A}$ (**d**), *TbRAP1*$^{F/2FQ}$ (**e**), *TbRAP1*$^{F/2FA}$ (**f**) and *TbRAP1*$^{F/2FL}$ (**g**) cells. **h-l** Western analyses were performed to compare expression of F2H-tagged *Tb*RAP1 proteins. Whole cell lysates were prepared from *TbRAP1*$^{F/\Delta RRM}$ and *TbRAP1*$^{F/\Delta MybL}$ (**h**), *TbRAP1*$^{F/2FQ}$ (**i**), *TbRAP1*$^{F/2FL}$ (**j**), *TbRAP1*$^{F/2FA}$ (**k**), and *TbRAP1*$^{F/2FA\&5A}$ (**l**) cells. The whole cell lysate from *TbRAP1*$^{F2H+/-}$ was used as a control. The HA antibody 12CA5 (MSKCC Antibody & Bioresource Core) and tubulin antibody TAT-1 [49] were used. Molecular marker was run on the left lane in each gel and their sizes are indicated on the left. **m** ChIP experiments using the HA antibody 12CA5, a *Tb*TRF rabbit antibody [30], and IgG were done in *TbRAP1*$^{F2H+/-}$ cells and Cre-induced (for 30 h) *TbRAP1*$^{F/2FA}$ cells. Average and standard deviation were calculated from three independent experiments. The *p* value of two-sided unpaired *t*-test is shown for 12CA5 ChIP (*TbRAP1*$^{F/2FA}$ vs *TbRAP1*$^{F2H+/-}$). Error bars represent standard deviation. Source data are provided as a Source Data file.

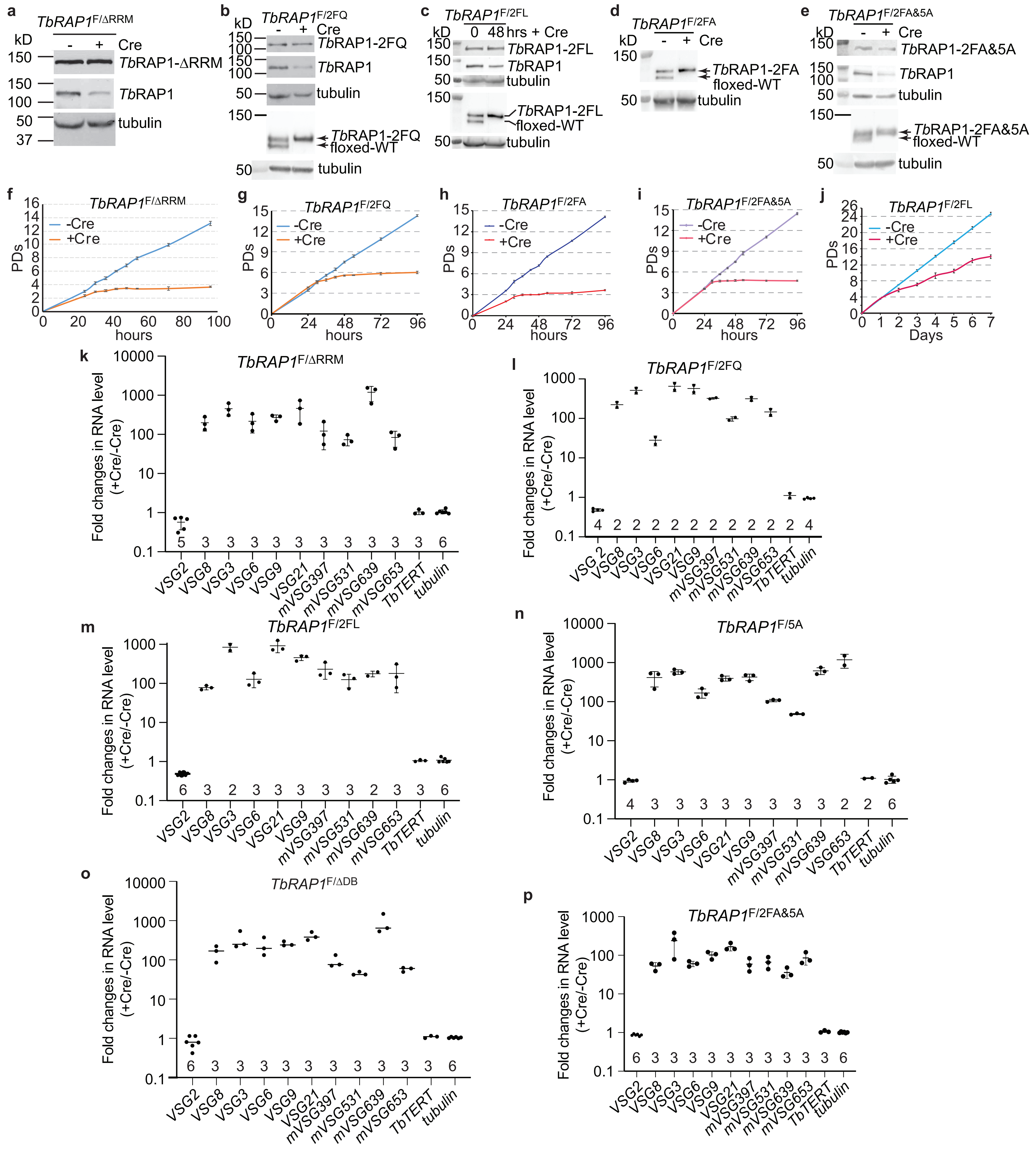

a
TbRAP1F/ΔRRM
kD
Cre
TbRAP1-ΔRRM
TbRAP1
tubulin
b
TbRAP1F/2FQ
TbRAP1-2FQ
floxed-WT
c
TbRAP1F/2FL
hrs + Cre
TbRAP1-2FL
d
TbRAP1F/2FA
TbRAP1-2FA
e
TbRAP1F/2FA&5A
TbRAP1-2FA&5A
-Cre
+Cre
PDs
hours
Days
Fold changes in RNA level (+Cre/-Cre)
TbRAP1F/2FL
TbRAP1F/5A
TbRAP1F/ΔDB
VSG2
VSG8
VSG3
VSG6
VSG9
VSG21
mVSG397
mVSG531
mVSG639
mVSG653
VSG653
TbTERT
tubulin

Supplementary Fig. 5. *Tb*RAP1 RRM mutants disrupt *VSG* MAE. **a-e** Western analysis of protein extracted from *TbRAP1*$^{F/\Delta RRM}$ (**a**), *TbRAP1*$^{F/2FQ}$ (**b**), *TbRAP1*$^{F/2FL}$ (**c**), *TbRAP1*$^{F/2FA}$ (**d**), and *TbRAP1*$^{F/2FA\&5A}$ (**e**) cells before and after induction of Cre for 30 h using the HA antibody "HA probe", a *Tb*RAP1 rabbit antibody, and the tubulin antibody TAT-1 (top three rows). In (**b, c, d, e**), to separate *Tb*RAP1 mutants and the endogenous *Tb*RAP1, proteins were run on a 7.5% Tris polyacrylamide gel for 7 h and detected by the *Tb*RAP1 rabbit antibody (the fourth row in **b, c, e**). The associated tubulin loading control is shown at the bottom. Molecular weight markers are run in the left lane or marked by a bar and their sized are indicated on the left. **f-j** Growth curves of *TbRAP1*$^{F/\Delta RRM}$ (**f**), *TbRAP1*$^{F/2FQ}$ (**g**), *TbRAP1*$^{F/2FA}$ (**h**), *TbRAP1*$^{F/2FA\&5A}$ (**i**), and *TbRAP1*$^{F/2FL}$ (**j**) cells with and without Cre. Average values were calculated from three independent experiments. PDs, population doublings. **k-p** qRT-PCR of RNA levels of the active *VSG2*, several silent ES-linked *VSGs*, and chromosome internal *TbTERT* and *tubulin* in *TbRAP1*$^{F/\Delta RRM}$ (**k**), *TbRAP1*$^{F/2FQ}$ (**l**), *TbRAP1*$^{F/2FL}$ (**m**), *TbRAP1*$^{F/5A}$ (**n**), *TbRAP1*$^{F/\Delta DB}$ (**o**), and *TbRAP1*$^{F/2FA\&5A}$ (**p**) cells after a 30-48 h induction of Cre. Average and standard deviation were calculated from two to six independent experiments (exact number of samples is listed at the bottom of each column). Error bars represent standard deviation. Source data are provided as a Source Data file.

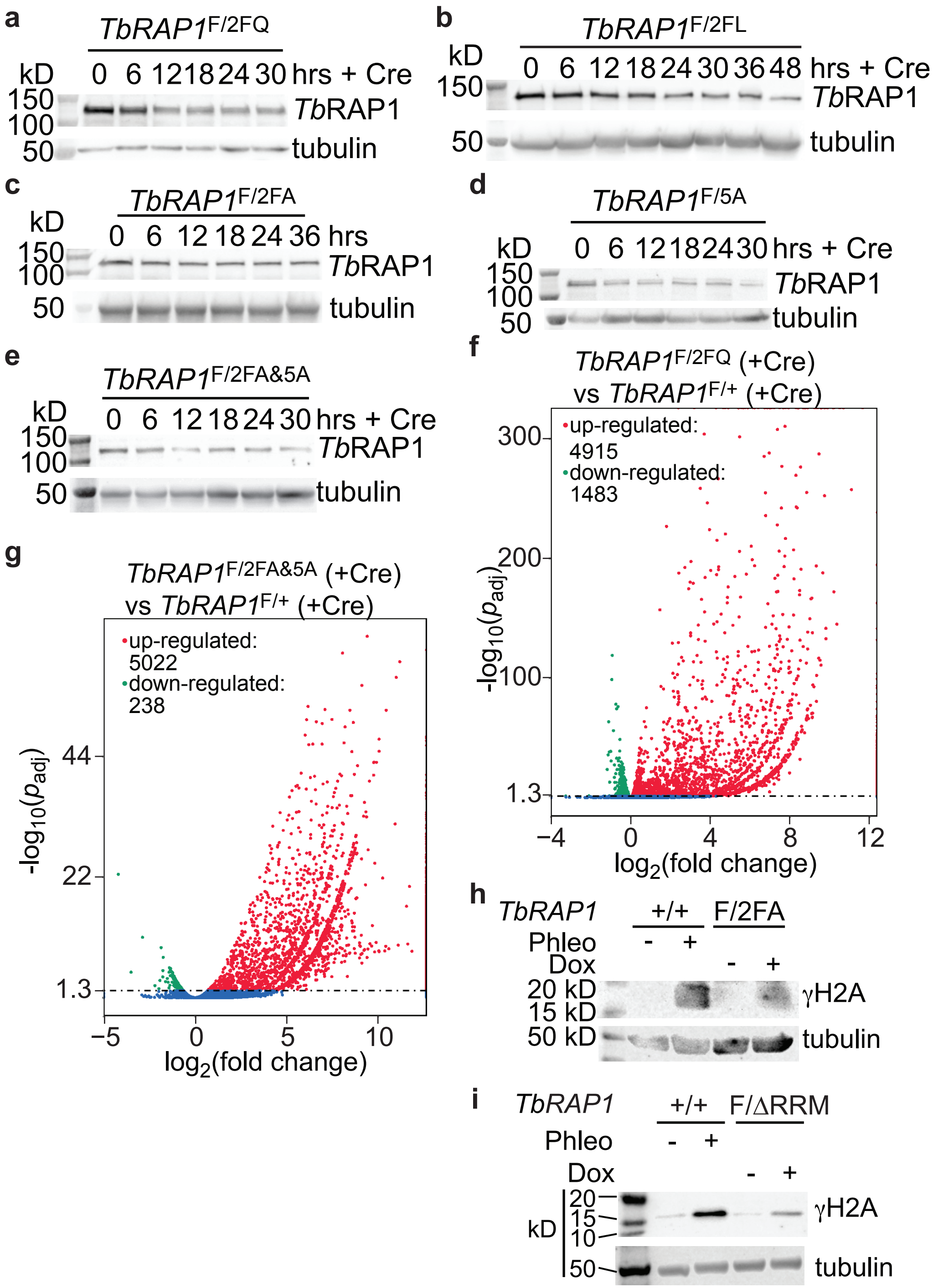
a
TbRAP1F/2FQ
kD
0 6 12 18 24 30 hrs + Cre
150
100
TbRAP1
50
tubulin
b
TbRAP1F/2FL
kD
0 6 12 18 24 30 36 48 hrs + Cre
150
TbRAP1
50
tubulin
c
TbRAP1F/2FA
kD
0 6 12 18 24 36 hrs
150
100
TbRAP1
50
tubulin
d
TbRAP1F/5A
kD
0 6 12 18 24 30 hrs + Cre
150
100
TbRAP1
50
tubulin
e
TbRAP1F/2FA&5A
kD
0 6 12 18 24 30 hrs + Cre
150
100
TbRAP1
50
tubulin
f
TbRAP1F/2FQ (+Cre) vs TbRAP1F/+ (+Cre)
up-regulated: 4915
down-regulated: 1483
-log10(padj)
300
200
100
1.3
−4
0
4
8
12
log2(fold change)
g
TbRAP1F/2FA&5A (+Cre) vs TbRAP1F/+ (+Cre)
up-regulated: 5022
down-regulated: 238
-log10(padj)
44
22
1.3
−5
0
5
10
log2(fold change)
h
TbRAP1
+/+
F/2FA
Phleo
-
+
Dox
-
+
20 kD
15 kD
γH2A
50 kD
tubulin
i
TbRAP1
+/+
F/ΔRRM
Phleo
-
+
Dox
-
+
kD
20
15
10
γH2A
50
tubulin

Supplementary Fig. 6. *Tb*RAP1 RRM mutants are defective in *VSG* regulation and lead to an increased amount of telomeric DNA damage. **a-e** Western analyses of protein extracted from *TbRAP1*$^{F/2FQ}$ (**a**), *TbRAP1*$^{F/2FL}$ (**b**), *TbRAP1*$^{F/2FA}$ (**c**), *TbRAP1*$^{F/5A}$ (**d**), and *TbRAP1*$^{F/2FA\&5A}$ (**e**) cells before and after induction of Cre using a *Tb*RAP1 rabbit antibody and the tubulin antibody TAT-1. Molecular weight markers are run in the left lane and their sized are indicated on the left. **f, g** Volcano plots of genes up-regulated and down-regulated in *TbRAP1*$^{F/2FQ}$ (**f**) and *TbRAP1*$^{F/2FA\&5A}$ (**g**) cells compared to *TbRAP1*$^{F/+}$ cells after a 30-hr Cre induction. *P* values of Wald test were calculated in DESeq and were further adjusted using the Benjamini and Hochberg's approach. **h, i** Western analyses to examine the γH2A protein level in WT cells before and after phleomycin treatment (as a positive control) and in *TbRAP1*$^{F/2FA}$ (**h**) and *TbRAP1*$^{F/\Delta RRM}$ (**i**) cells before and after a 30 h Cre induction. A γH2A rabbit antibody and the tubulin antibody TAT-1 were used. Source data are provided as a Source Data file.

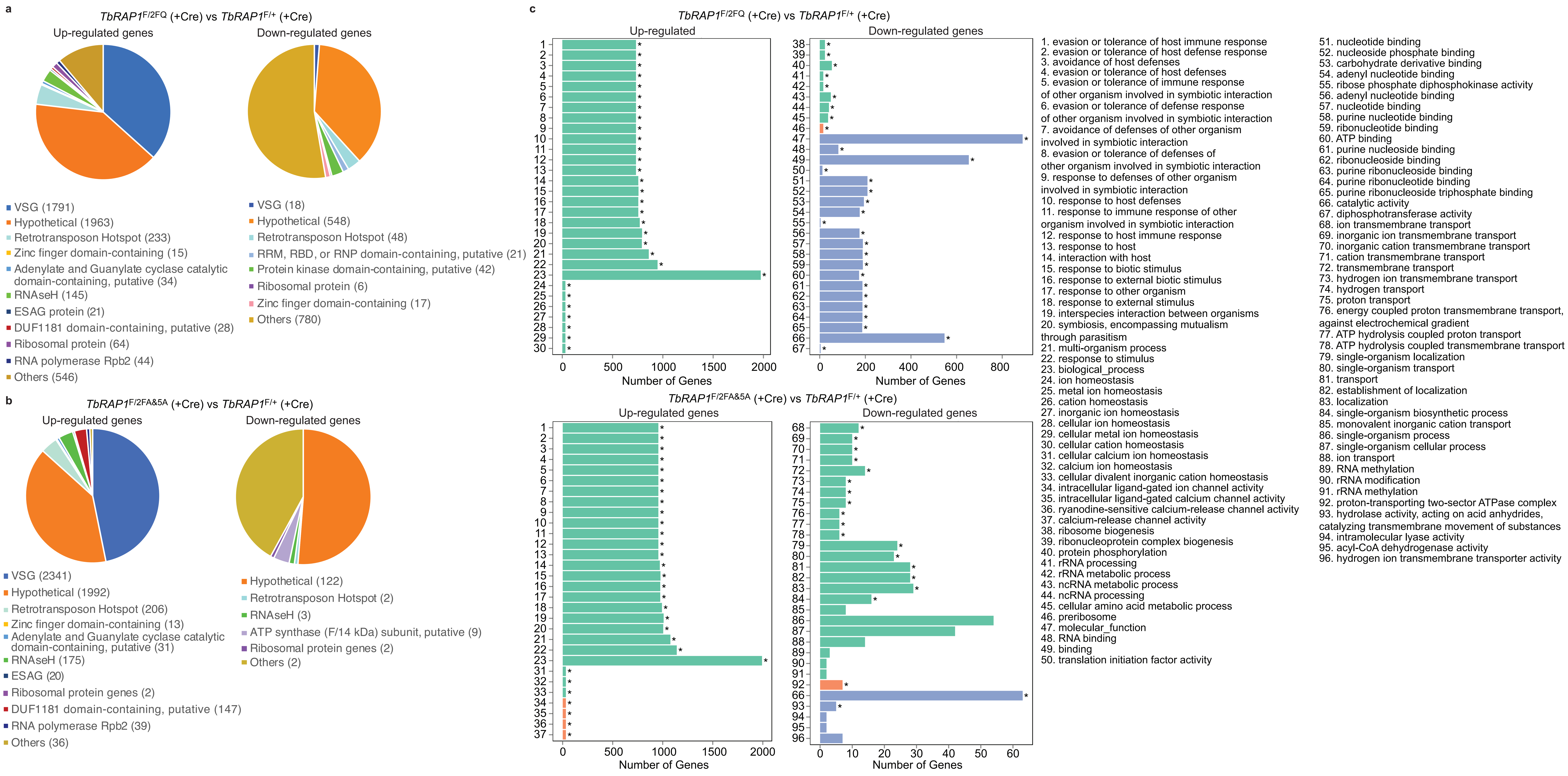
a
TbRAP1F/2FQ (+Cre) vs TbRAP1F/+ (+Cre)
Up-regulated genes
Down-regulated genes
VSG (1791)
Hypothetical (1963)
Retrotransposon Hotspot (233)
Zinc finger domain-containing (15)
Adenylate and Guanylate cyclase catalytic domain-containing, putative (34)
RNAseH (145)
ESAG protein (21)
DUF1181 domain-containing, putative (28)
Ribosomal protein (64)
RNA polymerase Rpb2 (44)
Others (546)
VSG (18)
Hypothetical (548)
Retrotransposon Hotspot (48)
RRM, RBD, or RNP domain-containing, putative (21)
Protein kinase domain-containing, putative (42)
Ribosomal protein (6)
Zinc finger domain-containing (17)
Others (780)
b
TbRAP1F/2FA&5A (+Cre) vs TbRAP1F/+ (+Cre)
Up-regulated genes
Down-regulated genes
VSG (2341)
Hypothetical (1992)
Retrotransposon Hotspot (206)
Zinc finger domain-containing (13)
Adenylate and Guanylate cyclase catalytic domain-containing, putative (31)
RNAseH (175)
ESAG (20)
Ribosomal protein genes (2)
DUF1181 domain-containing, putative (147)
RNA polymerase Rpb2 (39)
Others (36)
Hypothetical (122)
Retrotransposon Hotspot (2)
RNAseH (3)
ATP synthase (F/14 kDa) subunit, putative (9)
Ribosomal protein genes (2)
Others (2)
c
TbRAP1F/2FQ (+Cre) vs TbRAP1F/+ (+Cre)
Up-regulated
Down-regulated genes
Number of Genes
Number of Genes
TbRAP1F/2FA&5A (+Cre) vs TbRAP1F/+ (+Cre)
Up-regulated genes
Down-regulated genes
Number of Genes
Number of Genes
1. evasion or tolerance of host immune response
2. evasion or tolerance of host defense response
3. avoidance of host defenses
4. evasion or tolerance of host defenses
5. evasion or tolerance of immune response of other organism involved in symbiotic interaction
6. evasion or tolerance of defense response of other organism involved in symbiotic interaction
7. avoidance of defenses of other organism involved in symbiotic interaction
8. evasion or tolerance of defenses of other organism involved in symbiotic interaction
9. response to defenses of other organism involved in symbiotic interaction
10. response to host defenses
11. response to immune response of other organism involved in symbiotic interaction
12. response to host immune response
13. response to host
14. interaction with host
15. response to biotic stimulus
16. response to external biotic stimulus
17. response to other organism
18. response to external stimulus
19. interspecies interaction between organisms
20. symbiosis, encompassing mutualism through parasitism
21. multi-organism process
22. response to stimulus
23. biological_process
24. ion homeostasis
25. metal ion homeostasis
26. cation homeostasis
27. inorganic ion homeostasis
28. cellular ion homeostasis
29. cellular metal ion homeostasis
30. cellular cation homeostasis
31. cellular calcium ion homeostasis
32. calcium ion homeostasis
33. cellular divalent inorganic cation homeostasis
34. intracellular ligand-gated ion channel activity
35. intracellular ligand-gated calcium channel activity
36. ryanodine-sensitive calcium-release channel activity
37. calcium-release channel activity
38. ribosome biogenesis
39. ribonucleoprotein complex biogenesis
40. protein phosphorylation
41. rRNA processing
42. rRNA metabolic process
43. ncRNA metabolic process
44. ncRNA processing
45. cellular amino acid metabolic process
46. preribosome
47. molecular_function
48. RNA binding
49. binding
50. translation initiation factor activity
51. nucleotide binding
52. nucleoside phosphate binding
53. carbohydrate derivative binding
54. adenyl nucleotide binding
55. ribose phosphate diphosphokinase activity
56. adenyl nucleotide binding
57. nucleotide binding
58. purine nucleotide binding
59. ribonucleotide binding
60. ATP binding
61. purine nucleoside binding
62. ribonucleoside binding
63. purine ribonucleoside binding
64. purine ribonucleotide binding
65. purine ribonucleoside triphosphate binding
66. catalytic activity
67. diphosphotransferase activity
68. ion transmembrane transport
69. inorganic ion transmembrane transport
70. inorganic cation transmembrane transport
71. cation transmembrane transport
72. transmembrane transport
73. hydrogen ion transmembrane transport
74. hydrogen transport
75. proton transport
76. energy coupled proton transmembrane transport, against electrochemical gradient
77. ATP hydrolysis coupled proton transport
78. ATP hydrolysis coupled transmembrane transport
79. single-organism localization
80. single-organism transport
81. transport
82. establishment of localization
83. localization
84. single-organism biosynthetic process
85. monovalent inorganic cation transport
86. single-organism process
87. single-organism cellular process
88. ion transport
89. RNA methylation
90. rRNA modification
91. rRNA methylation
92. proton-transporting two-sector ATPase complex
93. hydrolase activity, acting on acid anhydrides, catalyzing transmembrane movement of substances
94. intramolecular lyase activity
95. acyl-CoA dehydrogenase activity
96. hydrogen ion transmembrane transporter activity

Supplementary Fig. 7. Classification of genes affected in Cre-induced *TbRAP1*$^{F/2FQ}$ (**a**) and *TbRAP1*$^{F/2FA\&5A}$ (**b**) cells. Distribution (percent of total affected genes) of different types of genes (based on TriTrypDB annotation) is shown. The number of genes affected in each category is listed in the parentheses. **c** GO term analysis of genes affected in Cre-induced *TbRAP1*$^{F/2FQ}$ (top) and *TbRAP1*$^{F/2FA\&5A}$ (bottom) cells. *P* values in hypergenometric test were calculated and corrected. GO terms with $p<0.05$ are considered significantly enriched in differential expression, which is marked with an asterisk (*).

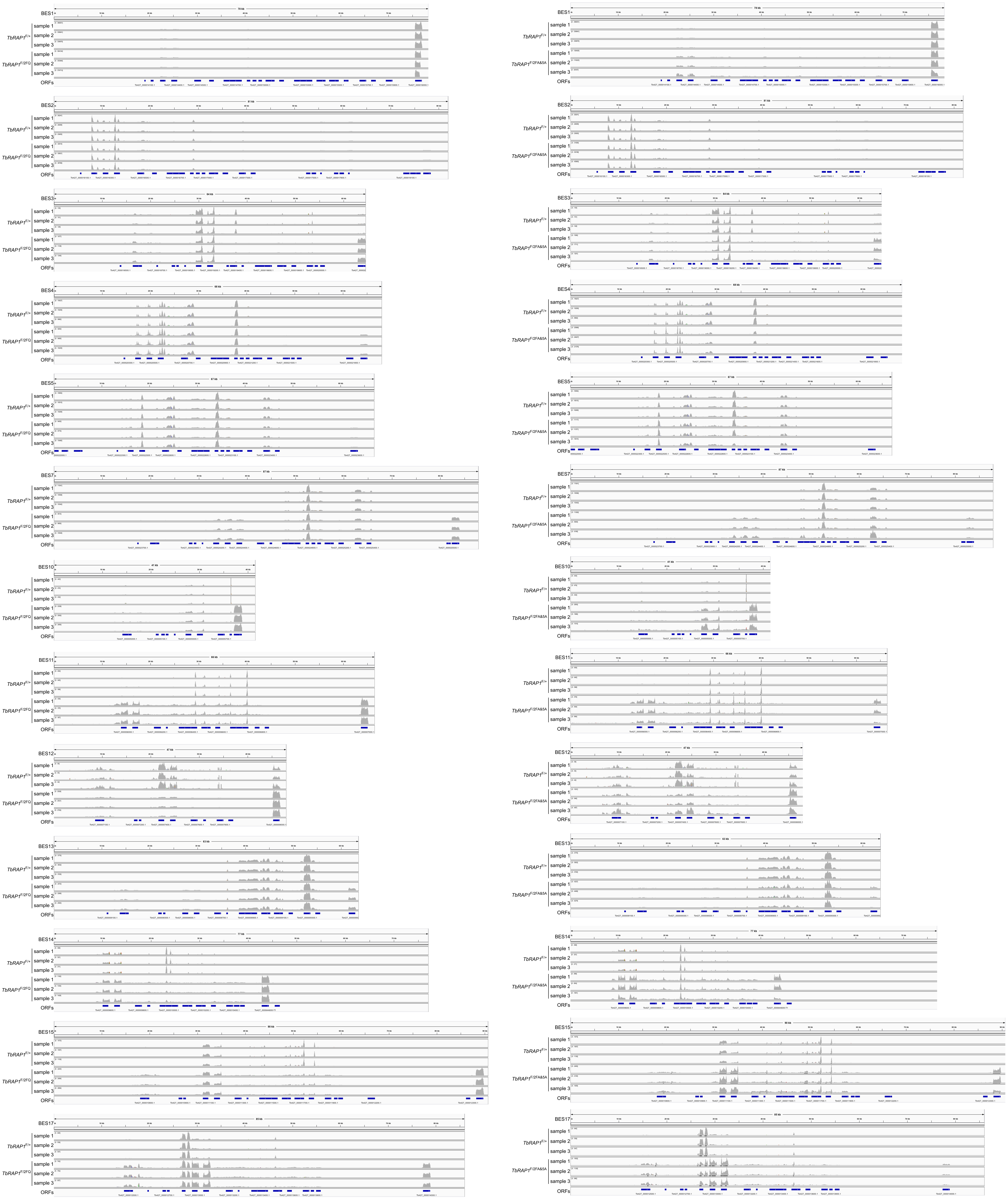

BES1
BES2
BES3
BES4
BES5
BES7
BES10
BES11
BES12
BES13
BES14
BES15
BES17
sample 1
sample 2
sample 3
TbRAP1F/+
TbRAP1F/2FQ
TbRAP1F/2FA&5A
ORFs

Supplementary Fig. 8. RNA sequence read coverage in all bloodstream form *VSG* ESs (BESs) in Cre-induced *TbRAP1*$^{F/+}$ and *TbRAP1*$^{F/2FQ}$ cells (left) and in Cre-induced *TbRAP1*$^{F/+}$ and *TbRAP1*$^{F/2FA\&5A}$ cells (right). RNA samples were isolated from three independently induced cells in all three strains. RNA sequence reads were mapped to the *T. brucei* lister 427 genome TriTrypDB-45_TbruceiLister427_2018_Genome.fasta [8], which was obtained from the TriTrypDB. Read coverage for each sample was viewed in IGV. Each BES is labeled and the genes inside each BES are marked at the bottom. The whole BES region is shown with the promoter located on the left side. The y axis represents the number of RNA reads. The range is indicated on the left of each trace. BES1 is fully active in the cells analyzed.

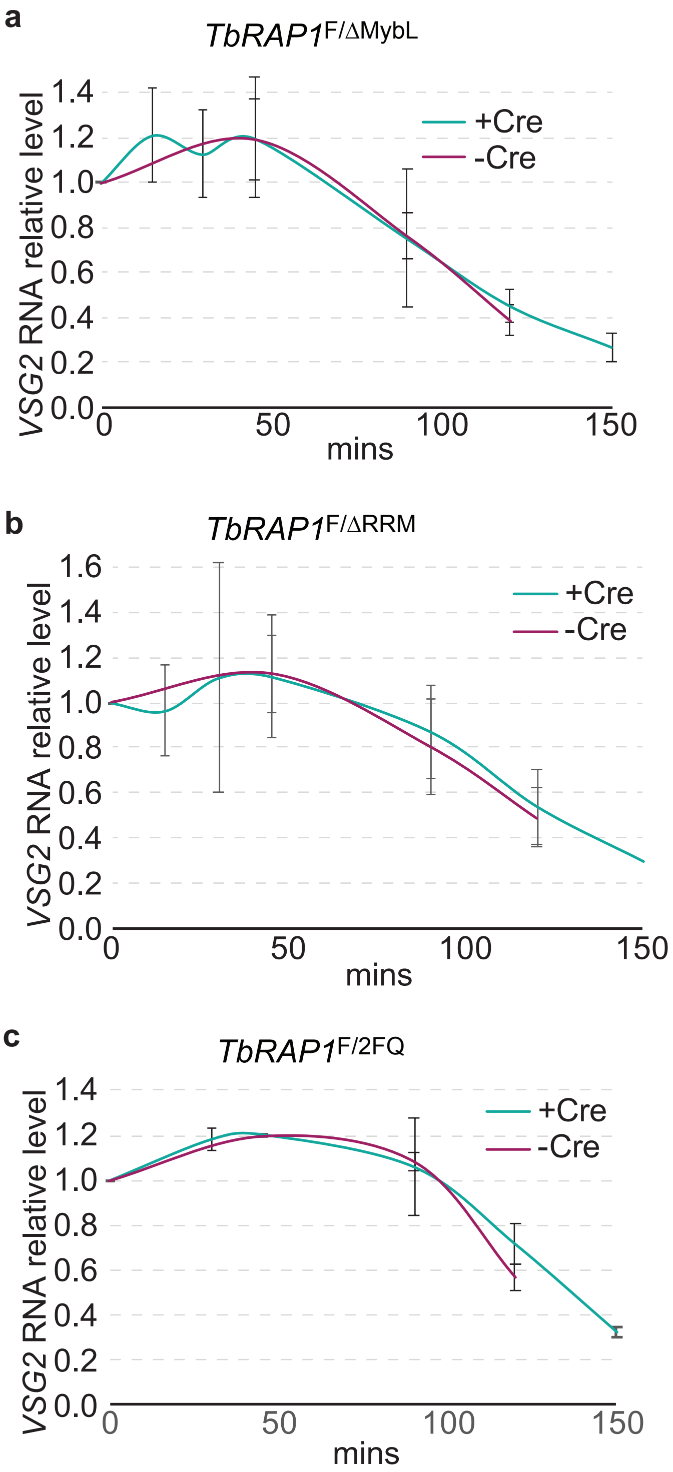
a
TbRAP1F/ΔMybL
VSG2 RNA relative level
1.4
1.2
1.0
0.8
0.6
0.4
0.2
0.0
0
50
100
150
mins
+Cre
-Cre
b
TbRAP1F/ΔRRM
VSG2 RNA relative level
1.6
1.4
1.2
1.0
0.8
0.6
0.4
0.2
0.0
0
50
100
150
mins
+Cre
-Cre
c
TbRAP1F/2FQ
VSG2 RNA relative level
1.4
1.2
1.0
0.8
0.6
0.4
0.2
0.0
0
50
100
150
mins
+Cre
-Cre

Supplementary Fig. 9. The *VSG2* RNA half-life is not affected by the *Tb*RAP1 RRM mutants. The *VSG2* RNA levels in *TbRAP1*$^{F/\Delta MybL}$ (**a**), *TbRAP1*$^{F/\Delta RRM}$ (**b**), and *TbRAP1*$^{F/2FQ}$ (**c**) cells after 29 h of induction of Cre at different time points (min) after adding Actinomycin D were estimated by qRT-PCR and normalized against the level at 0 min. Average and standard deviation were calculated from three (**a**), two to five (**b**), and two to three (**c**) independent experiments. Source data are provided as a Source Data file.

Supplementary Table 1. Statistics of NMR structure calculation

**Distance and dihedral restraints**	
Total number of NOE	1960
intra-residue ($\lvert i-j \rvert=0$)	845
sequential ($\lvert i-j \rvert=1$)	423
medium-range ($1<\lvert i-j \rvert<5$)	222
long-range ($\lvert i-j \rvert >4$)	470
Number of hydrogen bond restraints	38
Number of dihedral angle restraints	48
**Structure calculation**	
Violations	
Distance restraints (Å)	0.033 ± 0.002
Dihedral restraints (^{o})	0.67 ± 0.14
No. of dihedral angles with violation > 5°	0
No. of distant restraints with violation > 0.5Å	0
Average deviation from idealized geometry	
Bond length (Å)	0.006
Bond angle (^{o})	0.786
Improper (^{o})	0.495
*Average RMSD to mean structure (Å) **	
backbone	0.63
heavy	1.08
Average Ramachandran plot statistics (%)	
Most favoured	83.6
Additional allowed	14.1
Generously allowed	2.3
Disallowed	0

* RMSD of the RNA-Recognition Motif (653-727)

Supplementary Table 2. List of NMR titration and EMSA substrate sequences

Name	Sequence (5' to 3')
34-*VSG*-UTR[a]	AAAACUUUUUGAUAUAUUUUAACACCAAAACCAG
$(UUAGGG)_2$	UUAGGGUUAGGG
35-random	ACAGACCACUACAAGAUACACAGUACAACCAACCA
16-*VSG*-UTR[a]	UGAUAUAUUUUAACAC
170-*VSG*-UTR[a]	GGGCGAAUUGGGUACCGGGCCCCCCCUCGAGGUCGACGGUAUCGAUAAGCUUGAUAUCGAAUUCAAUUCAUUUCCCCCCUCAAAUUUCCCCCCUCCUUUUAAAAUUUUCCUUGCUACUUGAAAACUUUUUGAUAUAUUUUAACACCAAAACCAGCCGAGAUUUGGGAUCC
170-no-*VSG*	GGGCGAAUUGGGUACCGGGCCCCCCCUCGAGGUCGACGGUAUCGAUAAGCUUGAUAUCGAAUUCAAUUCCAAAGAAGCCAUGCGCCGUGCAGAGCAGGAAAAAGCCACACGGGAACUCCUCCUGCAACAAACUUCAGCACCACAUGCAUCAUCAGUUACAUCCGGGAUCC
35-*VSG*-UTR[a]	UUGCUACUUGAAAACUUUUUGAUAUAUUUUAACAC
100-ds(TTAGGG)	GAATTCGAGCCCGGGTTAGGGTTAGGGTTAGGGTTAGGGTTAGGGTTAGGGTTAGGGTTAGGGTTAGGGTTAGGGTTAGGGTTAGGGCCCCTCTAGAGTCGA
81-*VSG*-UTR[a]	GGGUAAUUUCCCCCCUCAAAUUUCCCCCCUCCUUUUAAAAUUUUCCUUGCUACUUGAAAACUUUUUGAUAUAUUUUAACAC
80-dsDNA	CGTGCAGAGCAGGAAAAAGCCACACGGGAACTCCTCCTGCAACAAACTTCAGCACCACATGCATCATCAGTTACATCCGG

[a] The consensus 9-mer and 16-mer sequences in *VSG* 3'UTRs [19,20] are underlined.

Supplementary Table 3. List of recombinant proteins.

Plasmid Name	Recombinant Protein	RAP1 Domain	Mutation	*E. coli* strain
pET32a-TrxA-His$_6$-3C	TrxA-His$_6$			ArcticExpress (DE3)
pET32a-TrxA-His$_6$-3C-*Tb*RAP1(639-761) [17]	TrxA-His$_6$-*Tb*RAP1$_{639-761}$	MybLike	WT, with a TrxA-His$_6$ tag	ArcticExpress (DE3)
pET32a-TrxA-His$_6$-3C-*Tb*RAP1(639-761) [17]	*Tb*RAP1$_{639-761}$	MybLike	WT, no tag	ArcticExpress (DE3)
pET32a-TrxA-His$_6$-3C-*Tb*RAP1(639-733) [17]	TrxA-His$_6$-*Tb*RAP1$_{639-733}$	RRM	WT, with a TrxA-His$_6$ tag	ArcticExpress (DE3)
pET32a-TrxA-His$_6$-3C-*Tb*RAP1(639-733) [17]	*Tb*RAP1$_{639-733}$	RRM	WT, no tag	ArcticExpress (DE3)
pET32a-TrxA-His$_6$-3C-*Tb*RAP1(639-761) 2FA&5A	TrxA-His$_6$-*Tb*RAP1$_{639-761}$2FA&5A	MybLike	F655A F694A 737RKRRR741 to 737AAAAA741, with a TrxA-His$_6$ tag	BL21 (DE3)
pET32a-TrxA-His$_6$-3C-*Tb*RAP1(639-761) 5A [17]	TrxA-His$_6$-*Tb*RAP1$_{639-761}$5A	MybLike	737RKRRR741 to 737AAAAA741, with a TrxA-His$_6$ tag	BL21 (DE3)
pET32a-TrxA-His$_6$-3C-*Tb*RAP1(639-761) 5A [17]	*Tb*RAP1$_{639-761}$5A	MybLike	737RKRRR741 to 737AAAAA741, no tag	BL21 (DE3)
pGEX-4T-2	GST			BL21 (DE3)
pGEX-4T-2-*Tb*RAP1(414-855) [17]	GST-*Tb*RAP1$_{414-855}$	Myb, MybLike, RCT	WT, with a GST tag	Rosetta (DE3)
pGEX-4T-2-*Tb*TRF [30]	GST-*Tb*TRF	*Tb*TRF full-length protein	WT, with a GST tag	BL21 (DE3)
pET32a-TrxA-His$_6$-3C-*Tb*RAP1(639-733) 2FQ	*Tb*RAP1$_{639-733}$ 2FQ	RRM	F655QF694Q No tag	ArcticExpress (DE3)

pET32a-TrxA-His$_6$-3C-*Tb*RAP1(639-733) 2FL	*Tb*RAP1$_{639-733}$ 2FL	RRM	F655LF694L No tag	ArcticExpress (DE3)
pET32a-TrxA-His$_6$-3C-*Tb*RAP1(639-733) 2FA	*Tb*RAP1$_{639-733}$ 2FA	RRM	F655AF694A No tag	ArcticExpress (DE3)

All RAP1 fragments used in NMR titration are free of tags. Most RAP1 fragments used in EMSA are free of tags, as labeled in figures.

Supplementary Table 4. **List of primers used in qRT-PCR and RNA-CLIP**

Gene/locus	Primer	Sequence (5' - 3')
VSG2	OBL-VSG221-FW1	GTCCTAGCCCAAGTTCTTC
	OBL-VSG221-BW1	GCTGTTGCAGTAGCTGTTAC
VSG3	OBL-VSG224-RT-FW	CAGTCTTGTGCGCACTAGCT
	OBL-VSG224-RT-BW	ATGCTGCTGCTGCTGTTACC
VSG6	OBL-VSG121-RT-FW	CAAGTTTGAGACGTGGGAGC
	OBL-VSG121-RT-BW	AGATCGGCTGCTATTGCTGC
VSG9	OBL-VSGVO2-RT-FW	AGCTTATCTAGCAGACGCCG
	OBL-VSGVO2-RT-BW	TCCTCCCATTTCCTCCGATC
VSG21	OBL-VSG21-FW	ACATGCGTCACATAACGCGG
	OBL-VSG21-RT-BW	TTGTTTGCTGGCCTGCTAGC
VSG8	OBL-VSG1.8-FW	CTACCAATTGCAGGGCTCAC
	OBL-VSG1.8-RT-BW	TTACGGCCAGCAGCTTGGAT
mVSG397	OBL-mVSG397-FW	GATACCAAGCAGTACTGGCC
	OBL-mVSG397-RT-BW	GCTTGTGCCTGTTGTTCCAG
mVSG531	OBL-mVSG531-FW	GGCTCACATTCTCTGAGGTG
	OBL-mVSG531-RT-BW	TTCCTTCTTGAGCGCTGCG
mVSG639	OBL-mVSG639-FW	CACGACTATCGCGGTACTC
	OBL-mVSG639-RT-BW	TGTCGCGTTGTCCTCTGTTACA
mVSG653	OBL-mVSG653-FW	TTGCCGCTTACGCTAGCG
	OBL-mVSG653-RT-BW	CCTTAGAAGGTTGACTGTCGC
70 bp Active	OBL-BES1-70bp-FW	AGTGTTGTGAGGGTGTGTATATACAA
	OBL-BES1-70bp-BW	TTATTATCATTACTACTCTTATTATAATACTCGC
70 bp Inactive	OBL-Telo65-70bp rep-FW	GTTGTGATAGTGTATATACGAATATTATAATAAGAGT
	OBL-Telo65-70bp rep-BW	CTCACAACACTCCCCTATTATTATTATTATTATTATT
ES1 promoter	OBL-BES1-unique-qPCR-FW	GTCACCGAGCTGCAAGAAC

	OBL-BES1-unique-qPCR-RW	GCCTTCCATCTGTTGCTGC
SNAP50	SNAP 50 RT FW2	CTATGAAGATTTGACGGCACCC
	SNAP 50 RT BW2	CTCGTGTGGTCATCTCGATCAA
rRNA	OBL-rDNA-RTPCR-1	ACGGAATGGCACCACAAGAC
	OBL-rDNA-RTPCR-2	GTCCGTTGACGGAATCAACC
Tubulin	OBL-bTubulin-RT-FW	ATCTTTGGACAGTCTGGCGC
	OBL-bTubulin-RT-BW	TTCCACAAGTTGGTGCACGG
TbTERT	OBL-tbTERT-RT-FW1	ATCGGTTTGCAGCTGCGTTG
	OBL-tbTERT-RT-BW1	TGCGGATGGTCTCCATCCAT
PKAC1	OBL-PKAC1-RT-FW1	CCAAGCCTGACACATCGG
	OBL-PKAC1-RT-BW1	GAAGGGGTGTGACAACTCC
