# Supplementary material for: The RRM-mediated RNA binding activity in T. brucei RAP1 is essential for VSG monoallelic expression
Source: Nat Commun. 2023 Mar 22;14:1576. doi: 10.1038/s41467-023-37307-0 (PMC10033678; doi:10.1038/s41467-023-37307-0)
Supplement: Supplementary file 3 — Reporting Summary [file 41467_2023_37307_MOESM3_ESM.pdf]

nature portfolio

Corresponding author(s): Bibo Li, Yanxiang Zhao

Last updated by author(s): Oct 16, 2022

Reporting Summary

Nature Portfolio wishes to improve the reproducibility of the work that we publish. This form provides structure for consistency and transparency in reporting. For further information on Nature Portfolio policies, see our Editorial Policies and the Editorial Policy Checklist.

Statistics

For all statistical analyses, confirm that the following items are present in the figure legend, table legend, main text, or Methods section.

n/a	Confirmed	
☐	☒	The exact sample size (*n*) for each experimental group/condition, given as a discrete number and unit of measurement
☐	☒	A statement on whether measurements were taken from distinct samples or whether the same sample was measured repeatedly
☐	☒	The statistical test(s) used AND whether they are one- or two-sided<br>*Only common tests should be described solely by name; describe more complex techniques in the Methods section.*
☒	☐	A description of all covariates tested
☒	☐	A description of any assumptions or corrections, such as tests of normality and adjustment for multiple comparisons
☐	☒	A full description of the statistical parameters including central tendency (e.g. means) or other basic estimates (e.g. regression coefficient) AND variation (e.g. standard deviation) or associated estimates of uncertainty (e.g. confidence intervals)
☐	☒	For null hypothesis testing, the test statistic (e.g. *F*, *t*, *r*) with confidence intervals, effect sizes, degrees of freedom and *P* value noted<br>*Give P values as exact values whenever suitable.*
☒	☐	For Bayesian analysis, information on the choice of priors and Markov chain Monte Carlo settings
☒	☐	For hierarchical and complex designs, identification of the appropriate level for tests and full reporting of outcomes
☒	☐	Estimates of effect sizes (e.g. Cohen's *d*, Pearson's *r*), indicating how they were calculated

Our web collection on statistics for biologists contains articles on many of the points above.

Software and code

Policy information about availability of computer code

Data collection	ImageQuant (v8.2), SoftWorx (v7.0), NMRPipe (v11.4)
Data analysis	MS Excel (v16), GraphPad Prism (v8.0.1 & v9.5), NMRFAM-Sparky (v1.470), CCPN (v3.1.1), CNSsolve (v1.3), Xplor-NIH (v3.3)

For manuscripts utilizing custom algorithms or software that are central to the research but not yet described in published literature, software must be made available to editors and reviewers. We strongly encourage code deposition in a community repository (e.g. GitHub). See the Nature Portfolio guidelines for submitting code & software for further information.

Data

Policy information about availability of data

All manuscripts must include a data availability statement. This statement should provide the following information, where applicable:

- Accession codes, unique identifiers, or web links for publicly available datasets
- A description of any restrictions on data availability
- For clinical datasets or third party data, please ensure that the statement adheres to our policy

The coordinates of the TbRAP1 RRM structures generated in this study have been deposited in the Protein Data Bank (PDB ID 7XRW: https://www.rcsb.org/structure/unreleased/7XRW) and BMRB (ID 36489: https://bmrb.io/data_library/summary/index.php?bmrbId=36489). The NMR titration data generated in this study have been deposited at BMRB (ID 51819: https://bmrb.io/data_library/summary/index.php?bmrbId=51819). The RNAseq data generated during this study are available at Geo repository (GSE193394: https://www.ncbi.nlm.nih.gov/geo/query/acc.cgi?acc=GSE193394). Source data are provided with this paper.

Human research participants

Policy information about studies involving human research participants and Sex and Gender in Research.

Reporting on sex and gender	N/A
Population characteristics	N/A
Recruitment	N/A
Ethics oversight	N/A

Note that full information on the approval of the study protocol must also be provided in the manuscript.

Field-specific reporting

Please select the one below that is the best fit for your research. If you are not sure, read the appropriate sections before making your selection.

☒ Life sciences ☐ Behavioural & social sciences ☐ Ecological, evolutionary & environmental sciences

For a reference copy of the document with all sections, see nature.com/documents/nr-reporting-summary-flat.pdf

Life sciences study design

All studies must disclose on these points even when the disclosure is negative.

Sample size	All samples were prepared from different biological replications. No sample size calculation was performed. By convention, we included at least two independent samples in each measurement to ensure reproducibility. Positive and negative controls are included in every tests so these are repeated more than a couple of times. Most measurements were done using at least three or more samples. However, since several point mutations were extensively analyzed, two samples of domain deletion mutants were included for selected analyses. Similarly, several silent VSG genes were analyzed for their RNA levels in various mutants, while selected VSG genes were examined twice at selected time points.
Data exclusions	One set of qRT-PCR results from TbRAP1-2FA&5A mutants were excluded. Two people performed the same experiment, each did the same set of experiments twice. Three sets of data gave consistent results while the last set shows as an outlier. With more detailed investigation, we found that the last set of data was generated when the cells were not fully recovered from thawing. Therefore, we excluded the last set of data in the final calculation.
Replication	Changes were evaluated in at least two sets of experiments and the trend of changes was found to be all consistent.
Randomization	Samples were allocated into experimental groups based on genotypes of various mutants and WT controls. We confirmed each strains genotype by Southern, PCR, and western analyses. For each genotype, at least two independent clones were randomly selected for subsequent phenotype analyses to ensure reproducibility.
Blinding	Control group (frequently WT cells) and test group (usually a mutant) are cultured with different antibiotics, as mutants need to be selected by various antibiotics to ensure that their genotypes do not drift. Therefore, it is impossible to blind the investigators about the group allocation when experiments were performed. However, data collection was performed independently from data analyses. Investigators who collected the data were not aware of the results at the time of performing experiments.

Reporting for specific materials, systems and methods

We require information from authors about some types of materials, experimental systems and methods used in many studies. Here, indicate whether each material, system or method listed is relevant to your study. If you are not sure if a list item applies to your research, read the appropriate section before selecting a response.

Materials & experimental systems

n/a	Involved in the study
☐	☒ Antibodies
☒	☐ Eukaryotic cell lines
☒	☐ Palaeontology and archaeology
☒	☐ Animals and other organisms
☒	☐ Clinical data
☒	☐ Dual use research of concern

Methods

n/a	Involved in the study
☒	☐ ChIP-seq
☒	☐ Flow cytometry
☒	☐ MRI-based neuroimaging

Antibodies

Antibodies used

HA antibody 12CA5 was purchased from Memorial Sloan Kettering Cancer Center Antibody and Bioresource Core Facility (no catalog number). VSG2 monoclonal antibody (IgM) was purchased from Memorial Sloan Kettering Cancer Center Antibody and Bioresource Core Facility (clone 13A8). HA antibody "HA Probe" was purchased from Santa Cruz Biotechnology, Inc. (Cat no. sc-7392). Antibodies recognizing TbRAP1, TbTRF, gammaH2A, tubulin, were custom raised and characterized in previously published papers (references have been given in the manuscript).

Validation

HA antibody 12CA5 has been used in western, IP, IF and ChIP experiments and published in previous studies: Benmerzouga et al. 2013. Mol. Micro. 87:196; Jehi et al. 2014. Cell Res. 24:870; Jehi et al. 2016. PLoS One 11:e0156746; Nanavaty et al. 2017. NAR 45:5784; Afrin et al. 2020. mSphere 5: e00027; Afrin et al. 2020. Sci. Adv. 6:eabc4065; Saha et al. 2021. NAR 49:5637; Rabbani et al. 2022. NAR 50:2036. The HA Probe antibody has been used in western and published in previous studies: Afrin et al. 2020. mSphere 5: e00027; Afrin et al. 2020. Sci. Adv. 6:eabc4065; Saha et al. 2021. NAR 49:5637. TbTRF rabbit antibody has been used in western, IF, IP, ChIP experiments and published in previous studies: Li et al. 2005. MCB 25:5011; Yang et al. 2009. Cell 137:99; Pandya et al. 2013. NAR 41:7673; Jehi et al. 2014. Cell Res. 24:870; Jehi et al. 2014. NAR 42:12899; Jehi et al. 2016. PLoS One 11:e0156746; Afrin et al. 2020. mSphere 5: e00027; Afrin et al. 2020. Sci. Adv. 6:eabc4065; Saha et al. 2021. NAR 49:5637; Rabbani et al. 2022. NAR 50:2036. TbRAP1 rabbit antibody has been used in western, IF, IP, ChIP experiments and published in previous studies: Yang et al. 2009. Cell 137:99; Pandya et al. 2013. NAR 41:7673; Jehi et al. 2016. PLoS One 11:e0156746; Nanavaty et al. 2017. NAR 45:5784; Afrin et al. 2020. mSphere 5: e00027; Afrin et al. 2020. Sci. Adv. 6:eabc4065. VSG rabbit antibody (excluding the cross-reaction part) has been used in western and IF and published in previous studies: Yang et al. 2009. Cell 137:99; Pandya et al. 2013. NAR 41:7673; Afrin et al. 2020. mSphere 5: e00027; Afrin et al. 2020. Sci. Adv. 6:eabc4065; Saha et al. 2021. NAR 49:5637; Rabbani et al. 2022. NAR 50:2036. VSG monoclonal antibody has been used in VSG switching assay and published in previous studies: Jehi et al. 2014. Cell Res. 24:870; Jehi et al. 2014. NAR 42:12899; Jehi et al. 2016. PLoS One 11:e0156746; Nanavaty et al. 2017. NAR 45:5784; Afrin et al. 2020. Sci. Adv. 6:eabc4065; Rabbani et al. 2022. NAR 50:2036. GammaH2A antibody has been used in western, IF, and ChIP experiments and published in previous studies: Nanavaty et al. 2017. NAR 45:5784; Afrin et al. 2020. Sci. Adv. 6:eabc4065; Saha et al. 2021. NAR 49:5637; Rabbani et al. 2022. NAR 50:2036. Tubulin antibody TAT-1 has been used in western and published in previous studies: Nanavaty et al. 2017. NAR 45:5784; Afrin et al. 2020. mSphere 5: e00027; Afrin et al. 2020. Sci. Adv. 6:eabc4065; Saha et al. 2021. NAR 49:5637; Rabbani et al. 2022. NAR 50:2036. VSG3 and VSG6 antibodies (excluding the cross-reaction part) have been used in IF and published in previous studies: Yang et al. 2009. Cell 137:99; Rabbani et al. 2022. NAR 50:2036.
